# Handbook on Sanitation

**Published:** 1901-12

**Authors:** 


HANDBOOK ON SANITATION. A manual of theoretical and practical sanitation. For students and physicians; for health, sanitary, tenement-house, plumbing, factory, food and other inspectors; as well as for candidates for all municipal sanitary positions. By GEORGE M. PRICE, M. D., Medical Sanitary Inspector, Department of Health, New York City; Inspector New York Sanitary Aid Society of the 10th Ward, 1885; Manager Model Tenement-houses of the New York Tenement-house Building Company, 1888; Inspector New York State Tenement-house Commission, 1895. Duodecimo, pp. 329. Illustrated. New York: John Wiley & Sons. 1901. [Price, 1.50 net.]

Dr. Price has written a book the aim of which is to present to the student and physician, and more especially to the present or prospective sanitary inspector, the essentials of sanitation. There is, we believe, no other work of its kind published in this country. The first part of the volume is devoted to the principles of sanitation; the second section to the practice thereof. How practically the writer deals with this part of the topic may be gathered from such chapter headings as The tenement house problem; Sweat-shops; The smoke nuisance; Bakeries, etc. The concluding division is devoted to sanitary law.

The book is dedicated to Jacob A. Riis. M. J. F.

